# GRASP55 regulates the unconventional secretion and aggregation of mutant huntingtin

**DOI:** 10.1016/j.jbc.2022.102219

**Published:** 2022-07-01

**Authors:** Erpan Ahat, Sarah Bui, Jianchao Zhang, Felipe da Veiga Leprevost, Lisa Sharkey, Whitney Reid, Alexey I. Nesvizhskii, Henry L. Paulson, Yanzhuang Wang

**Affiliations:** 1 Department of Molecular, Cellular and Developmental Biology, University of Michigan, Ann Arbor, Michigan, USA; 2 Department of Pathology, University of Michigan School of Medicine, Ann Arbor, Michigan, USA; 3 Department of Neurology, University of Michigan School of Medicine, Ann Arbor, Michigan, USA; 4 Department of Computational Medicine and Bioinformatics, University of Michigan, Ann Arbor, Michigan, USA

**Keywords:** GRASP55, unconventional secretion, Golgi, huntingtin, secretory autophagy, aggregation, neurodegeneration, AGC, automatic gain control, BafA1, bafilomycin A1, BFA, brefeldin A, CFTR, cystic fibrosis transmembrane conductance regulator, CSF, cerebrospinal fluid, DMEM, Dulbecco's modified Eagle's medium, EBSS, Earle′s balanced salt solution, ER, endoplasmic reticulum, ERGIC, ER–Golgi intermediate compartment, GO, Gene Ontology, GRASP, Golgi reassembly stacking protein, HD, Huntington’s disease, Htt, huntingtin, IT, injection time, 55KO, GRASP55 KO, LAMP, lysosomal membrane–associated protein, LDH, lactate dehydrogenase, MEF, mouse embryonic fibroblast, N2A, Neuro2A, OA, okadaic acid, PFA, paraformaldehyde, SOD1, superoxide dismutase 1, STS, staurosporine, TCA, trichloroacetic acid, TDP-43, TAR DNA–binding protein 43, TMT, tandem mass tag, Tu, tunicamycin, TX-100, Triton X-100

## Abstract

Recent studies demonstrated that the Golgi reassembly stacking proteins (GRASPs), especially GRASP55, regulate Golgi-independent unconventional secretion of certain cytosolic and transmembrane cargoes; however, the underlying mechanism remains unknown. Here, we surveyed several neurodegenerative disease–related proteins, including mutant huntingtin (Htt-Q74), superoxide dismutase 1 (SOD1), tau, and TAR DNA–binding protein 43 (TDP-43), for unconventional secretion; our results show that Htt-Q74 is most robustly secreted in a GRASP55-dependent manner. Using Htt-Q74 as a model system, we demonstrate that unconventional secretion of Htt is GRASP55 and autophagy dependent and is enhanced under stress conditions such as starvation and endoplasmic reticulum stress. Mechanistically, we show that GRASP55 facilitates Htt secretion by tethering autophagosomes to lysosomes to promote autophagosome maturation and subsequent lysosome secretion and by stabilizing p23/TMED10, a channel for translocation of cytoplasmic proteins into the lumen of the endoplasmic reticulum–Golgi intermediate compartment. Moreover, we found that GRASP55 levels are upregulated by various stresses to facilitate unconventional secretion, whereas inhibition of Htt-Q74 secretion by GRASP55 KO enhances Htt aggregation and toxicity. Finally, comprehensive secretomic analysis identified novel cytosolic cargoes secreted by the same unconventional pathway, including transgelin (TAGLN), multifunctional protein ADE2 (PAICS), and peroxiredoxin-1 (PRDX1). In conclusion, this study defines the pathway of GRASP55-mediated unconventional protein secretion and provides important insights into the progression of Huntington’s disease.

Neurodegenerative diseases are characterized by increased neuronal cell death and decreased cognitive abilities of patients. Different diseases, such as Alzheimer’s disease, Parkinson’s disease, Huntington’s disease (HD), and amyotrophic lateral sclerosis, are caused by different protein-based aberrations that affect different pathways; yet all cause neuronal cell death by forming inclusion bodies (1, 2, 3). These include protein aggregates formed by hyperphosphorylated tau in Alzheimer’s disease (2), mutant superoxide dismutase 1 (SOD1) (4), and TAR DNA–binding protein 43 (TDP-43) in amyotrophic lateral sclerosis (5), and mutant huntingtin (Htt) in HD (6, 7). Many of these neurotoxic proteins have been detected in the cerebrospinal fluid (CSF) of patients with diseases, indicating that these proteins could be secreted from cells (8, 9, 10). How the secretion affects protein aggregation and toxicity in neurons, and whether secretion facilitates the spreading of toxic proteins to neighboring cells, remain elusive.

In the conventional secretory pathway, transmembrane and secretory proteins, such as the amyloid precursor protein, are synthesized in the endoplasmic reticulum (ER) and transported to the Golgi, from where they are targeted to their final destinations. However, many neurotoxic proteins, such as Htt, SOD1, α-synuclein, tau, and TDP-43, are synthesized as cytosolic proteins. Secretion of these proteins is independent of the traditional secretory pathway in which the Golgi functions as a trafficking center. For example, mutant Htt exists in two forms, an intracellular form in patient neurons and an extracellular form in CSF (8) exported through a not well-characterized unconventional secretory pathway (11, 12).

Little is known about the machinery that controls unconventional secretion, and the interplay between Golgi-dependent conventional trafficking and Golgi-independent unconventional secretion has not been explored. Although the unconventional pathway is independent of the Golgi, unconventional secretion of numerous cytosolic proteins reported so far requires the Golgi reassembly stacking protein of 55 kDa, GRASP55 (GORASP2) (13, 14, 15). GRASP55, and its homolog GRASP65 (GORASP1), was first characterized as key Golgi stacking proteins (16, 17). They both form transoligomers to link the Golgi cisternae into the unique stacked architecture (18). Knockdown of GRASP55 and GRASP65 by RNAi, or KO of both GRASPs by CRISPR–cas9, results in Golgi fragmentation (18, 19). GRASPs were first linked to unconventional secretion of acyl-CoA binding protein (AcbA/Acb1) in *Dictyostelium discoideum* and *Saccharomyces cerevisiae* (20, 21). In *Drosophila*, the GRASP protein is involved in unconventional trafficking of α-integrin at specific stages of fly development (22). In mammalian cells, GRASPs are required for unconventional trafficking of cystic fibrosis transmembrane conductance regulator (CFTR) and the cytokine interleukin-1β (23, 24). How GRASP55 regulates autophagosome-dependent unconventional secretion and whether GRASP55 is required for secretion of neurotoxic proteins such as Htt are unknown.

In this study, we report that mutant Htt is unconventionally secreted in a GRASP55-dependent manner. Htt secretion is elevated under stress conditions, including energy deprivation and nutrient starvation, inhibition of proteasomal degradation, and induction of ER stress, all of which also upregulate GRASP55 expression. GRASP55 facilitates Htt secretion through two actions: one is to accelerate autophagic flux by facilitating autophagosome and lysosome fusion, the other is to stabilize p23/TMED10, a protein channel in the ER–Golgi intermediate compartment (ERGIC) that translocates cytosolic proteins into the ERGIC lumen for unconventional secretion. Finally, we performed systematic proteomic and secretomic analysis of WT and GRASP55 KO (55KO) cells and identified a list of cytoplasmic proteins whose secretion depends on GRASP55.

## Results

### GRASP55 is required for unconventional secretion of mutant Htt

To determine if GRASP55 is required for the secretion of cytosolic proteins related to neurodegeneration, we expressed a number of neurotoxic proteins, including Htt exon 1 fragment with a long polyglutamine (Q74) stretch (GFP-Htt-Q74), WT SOD1 (GFP-SOD1), TDP-43 (TDP-43-GFP), and tau (FLAG-tau), in WT and 55KO HeLa cells we established earlier (19). For Htt, we chose the Htt exon 1 fragment because it has been shown that this fragment is harder to degrade, more toxic than full-length Htt, and detected in the CSF with an elevated concentration in HD (25, 26, 27, 28). At 48 h post-transfection, we cultured the cells in a serum-free medium for 4 h and determined the level of the corresponding cargo in the conditioned media by Western blot. A conventional secretory pathway cargo, clusterin, was used as a control. All these proteins were found in the conditioned media (Fig. S1, *A*–*D*), but Htt-Q74 secretion was the most robust and GRASP55 dependent (Fig. S1*A*). Therefore, we used GFP-Htt-Q74 as a main marker to study the mechanism of GRASP55-dependent unconventional secretion.

**Figure 1:**
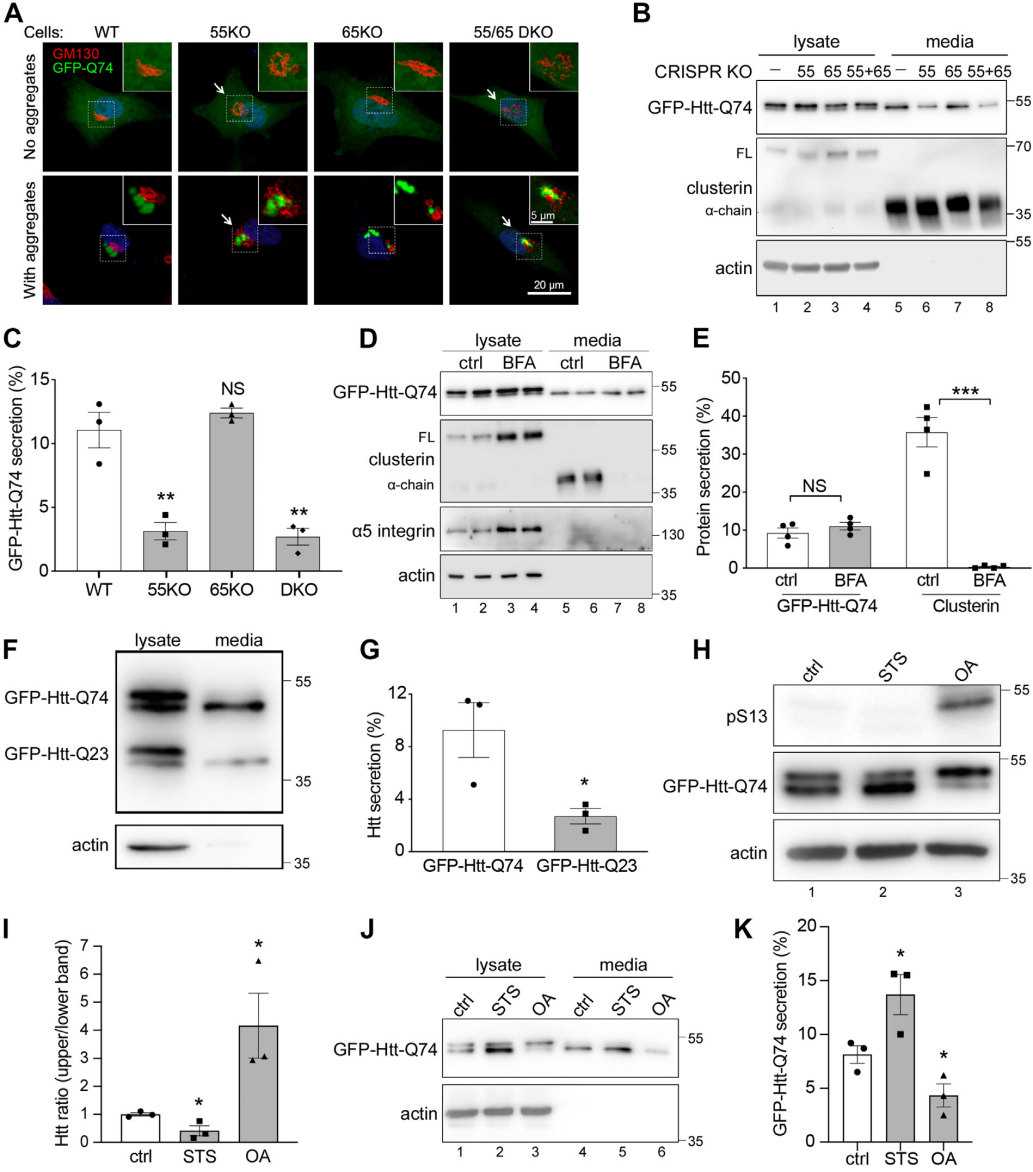
**GRASP55 is required for unconventional secretion of mutant huntingtin.***A*, GFP-Htt-Q74 aggregation induces Golgi fragmentation in GRASP55 KO (55KO) cells. WT and indicated GRASP KO HeLa cells expressing GFP-Htt-Q74 were stained for GM130 (*red*) and DNA (*blue*). Shown are example cells without (*upper panels*) or with (*lower panels*) Htt aggregates (*arrows*). *B*, Htt secretion requires GRASP55 but not GRASP65. Cell lysates and conditioned media of GFP-Htt-Q74–expressing WT and GRASP KO cells were analyzed by Western blot for Htt. Clusterin was used as a marker for conventional secretion. *C*, quantitation of Htt secretion in *B*. Htt secretion was calculated as Htt signal in conditioned medium/(Htt signal in conditioned medium + Htt signal in cell lysate) × 100%, with the loading ratio taken into consideration. *D*, Htt secretion is unaffected by the block of ER-to-Golgi trafficking. GFP-Htt-Q74–expressing WT cells were treated with or without 5 μg/ml BFA for 4 h, and Htt in the cell lysates and conditioned media were analyzed by Western blot. Note that BFA treatment reduced the level of clusterin but not Htt in the conditioned media. *E*, quantitation of Htt and clusterin secretion in *D*. *F*, Htt-Q74 is preferably secreted than Htt-Q23. WT cells were cotransfected by GFP-Htt-Q23 and GFP-Htt-Q74 for 24 h. Cells were then incubated in serum-free medium for 4 h followed by Western blot of Htt in the cell lysate and conditioned media. *G,* quantification of Htt-Q74 and Htt-Q23 in the conditioned media. *H*, Htt is phosphorylated. GFP-Htt-Q74–expressing cells were treated with 1 μM staurosporine (STS) or 1 μM okadaic acid (OA) for 4 h and analyzed by Western blotting for GFP and pS13-Htt. The pS13 antibody recognizes Htt phosphorylated at serine-13. *I*, quantification of Htt in H based on the GFP blot. *J*, dephosphorylated Htt is preferably secreted. Cells treated as in *H* were analyzed for GFP-Htt-Q74 secretion. *K*, quantification of *J*. Quantitation results are presented as mean ± SEM; statistical analysis was performed using Student’s *t* test. ∗*p* < 0.05; ∗∗*p* < 0.01; and ∗∗∗*p* < 0.001. BFA, brefeldin A; α-chain, mature clusterin; ER, endoplasmic reticulum; FL, full-length clusterin; GRASP, Golgi reassembly stacking protein; Htt, huntingtin.

Since both GRASP55 and GRASP65 have been shown to be involved in unconventional secretion (29), we expressed GFP-Htt-Q74 in WT, 55KO, 65KO, or double KO (both GRASP55 and GRASP65) HeLa cells. At 48 h post-transfection, about 90 to 95% cells contained soluble Htt in the cytosol, as indicated by the diffuse GFP signal. Another 5 to 10% of cells contained Htt aggregates that appeared as bright dots in the cytoplasm (Fig. 1*A*). Interestingly, these Htt aggregates were often found in the perinuclear region where the Golgi was localized. This prompted us to look at the effect of Htt expression on Golgi morphology. While soluble Htt did not seem to affect Golgi morphology, Htt aggregates induced Golgi defects as previously reported (30, 31), and the effect was more apparent in 55KO cells (Fig. 1*A*). For comparison, we also tested GFP-Htt-Q23 in parallel, which did not form aggregates or significantly affect the Golgi morphology (Fig. S1*E*). Similarly, expression of SOD1, TDP-43, or tau in WT cells had no significant impact on the Golgi structure (Fig. S1*F*).

Next, we tested whether Htt secretion is GRASP dependent. After transfection of GFP-Htt-Q74 in WT, 55KO, 65KO, and double KO cells for 24 h, we cultured the cells in serum-free medium for 4 h and determined the level of Htt in the conditioned media by Western blot. Htt was readily detectable in the conditioned media, and the level was significantly reduced in 55KO but not 65KO cells (Fig. 1, *B* and *C*), indicating that Htt secretion depends on GRASP55 but not GRASP65. Secretion and processing of clusterin, a cargo molecule secreted by the conventional secretory pathway, were not affected by GRASP KO (Fig. 1*B*). In this assay, actin and two additional cytosolic proteins, hexokinase (HK1) and glucokinase (GCK), were not detected in the conditioned media (Figs. 1*B* and S1*G*). In addition, GFP-Htt-Q74 expression did not cause cell death as determined by the lactate dehydrogenase (LDH) cytotoxicity assay (Fig. S1*H*). These results indicate that Htt was secreted rather than nonselectively released from dead cells. Taken together, GFP-Htt-Q74 expression causes Golgi defects especially in 55KO cells, whereas 55KO reduces GFP-Htt-Q74 secretion.

### Mutant Htt is selectively secreted through a Golgi-independent pathway

To confirm that Htt is secreted through an unconventional Golgi-independent pathway, we blocked ER-to-Golgi trafficking by brefeldin A (BFA) treatment. As shown in Figure 1, *D* and *E*, BFA treatment diminished clusterin in the conditioned media, indicating a block of conventional secretion. Htt secretion was unaffected by BFA treatment, confirming that it is secreted *via* a Golgi-independent unconventional secretory pathway.

The toxicity of Htt is associated with the length of the polyQ tract within the exon 1 fragment; Htt with more than ∼35 glutamines in the stretch is regarded as a disease mutant (6, 7). To determine whether the length of the polyQ tract affects Htt secretion, we coexpressed GFP-Htt-Q74 and GFP-Htt-Q23 in the same cells and compared their secretion. As shown in Figure 1, *F* and *G*, when GFP-Htt-Q74 and GFP-Htt-Q23 were coexpressed, the level of GFP-Htt-Q74 in the conditioned media was higher than that of GFP-Htt-Q23. This indicates that the selectivity for GFP-Htt-Q74 secretion relies on the polyQ tract. Indeed, when Q80-GFP (in the absence of Htt exon 1) was expressed in HeLa cells, it was secreted at a comparable rate as GFP-Htt-Q74 (∼10%), and Q80-GFP secretion was also robustly reduced in 55KO cells (Fig. S2, *A* and *B*).

Interestingly, Htt exhibited two bands, and only the lower band was detected in the conditioned media; we were curious about the difference between the two bands. Htt is highly modified by post-translational modifications, such as phosphorylation, acetylation, and cleavage (32). Among these, phosphorylation at S13 and S16 by TANK-binding kinase 1 or IκB kinase has been shown to reduce Htt aggregation and toxicity (33, 34). To determine whether the two bands represent different phosphorylated forms of Htt, we treated cells with a general serine/threonine kinase inhibitor, staurosporine (STS), or a general phosphatase inhibitor, okadaic acid (OA). STS treatment increased the intensity of the lower band, whereas OA treatment accumulated the upper band, suggesting that the upper band represents a phosphorylated form, whereas the lower band is dephosphorylated. This conclusion was confirmed using a phospho-specific antibody pS13 for Htt (34), which recognized only the upper band (Fig. 1, *H* and *I*). More importantly, STS treatment increased Htt secretion, whereas OA treatment reduced its secretion (Fig. 1, *J* and *K*), indicating that the dephosphorylated aggregate-prone form of Htt is preferentially secreted compared with the phosphorylated soluble form of Htt. Taken together, these results demonstrate that unconventional secretion is selective, at least to a certain degree, and may serve as a clearing mechanism for cells to expel toxic proteins.

### GFP-Htt-Q74 secretion is enhanced by cellular stress

It has been demonstrated that unconventional secretion is elevated under different stress conditions when the autophagy level is high (35), and that unconventional secretion of CFTR and Acb1 depends on autophagy or autophagy genes (36, 37). In addition, we discovered that GRASP55 functions as an energy sensor through O-GlcNAcylation to regulate autophagosome maturation. Under growth conditions, GRASP55 is O-GlcNAcylated and concentrated on the Golgi. Upon glucose starvation, GRASP55 is de-O-GlcNAcylated and relocated from the Golgi to the interface between autophagosomes and lysosomes, where it interacts with LC3 on autophagosomes and lysosomal membrane–associated protein 2 (LAMP2) on lysosomes and functions as a membrane tether to facilitate autophagosome–lysosome fusion (38). Similar responses of GRASP55 occur under amino acid starvation or when mammalian target of rapamycin activity is inhibited by Torin 1 (38, 39, 40). In addition, GRASP55 also interacts with Beclin-1 to facilitate the assembly and membrane association of the UVRAG phosphatidylinositol 3-kinase (PtdIns3K) complex, and thereby facilitates autophagosome maturation (39).

**Figure 2:**
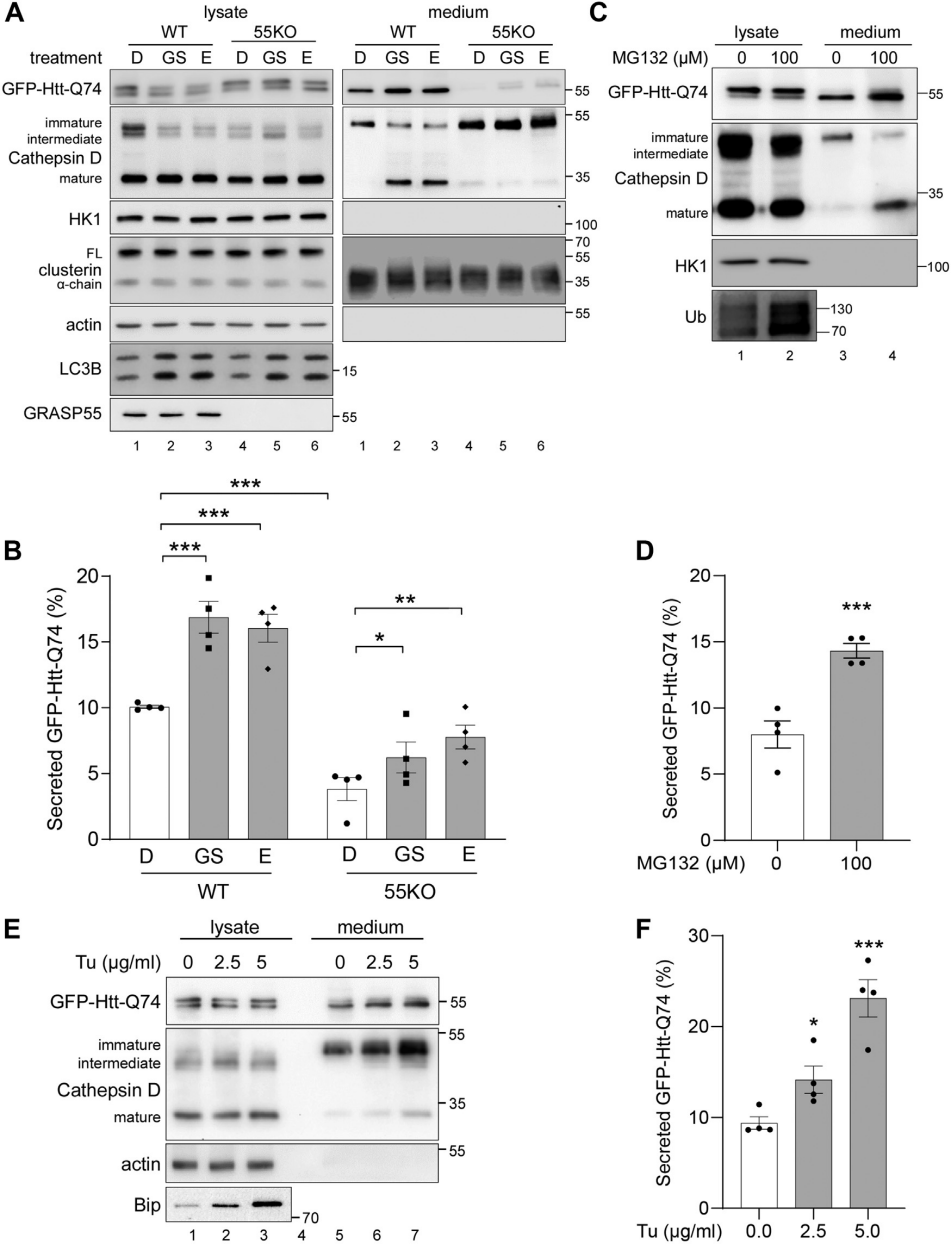
**Htt secretion is mediated by lysosome exocytosis, which is enhanced by various stress stimuli.***A*, Htt secretion is elevated by energy deprivation and nutrient starvation. WT and 55KO HeLa cells transfected with GFP-Htt-Q74 were incubated in serum-free DMEM (*D*), glucose-free medium (GS), or EBSS (*E*) for 4 h followed by Western blot of Htt and clusterin in the cell lysates and conditioned media. Cathepsin D was blotted to show lysosome exocytosis. Note that both starvation conditions enhanced Htt and mature cathepsin D secretion. *B*, quantification of Htt secretion in *A*. *C*, Htt secretion is enhanced by proteostasis stress. WT HeLa cells expressing GFP-Htt-Q74 were treated with 100 μM MG132 for 4 h followed by Western blot of Htt and cathepsin D in the cell lysates and conditioned media. Ubiquitin (Ub) was used as a control for MG132 treatment. Note that MG132 treatment increased the secretion of both Htt and mature cathepsin D. *D*, quantification of Htt secretion in *C*. *E*, Htt secretion is enhanced by ER stress. WT HeLa cells expressing GFP-Htt-Q74 were treated with indicated concentration of tunicamycin (Tu) for 4 h and analyzed by Western blot for indicated proteins. BiP was used as a marker for ER stress response. *F*, Quantification of *E*. Results are presented as mean ± SEM; statistical analysis was performed using Student’s *t* test. ∗*p* < 0.05; ∗∗*p* < 0.01. BiP, binding protein; DMEM, Dulbecco's modified Eagle's medium; EBSS, Earle′s balanced salt solution; ER, endoplasmic reticulum; Htt, huntingtin; 55KO, GRASP55 KO.

These observations prompted us to test the effect of energy deprivation and nutrient starvation on Htt secretion. As expected, both glucose starvation and amino acid deprivation (E, by incubating cells in Earle′s balanced salt solution [EBSS]) increased the level of Htt in the conditioned media compared with Dulbecco's modified Eagle's medium (DMEM) (D) (Fig. 2, *A* and *B*). A similar effect was observed in 55KO cells, although the overall secretion was significantly lower than WT cells. In addition to starvation, inhibiting proteasomal degradation with MG132, or inducing ER stress with tunicamycin (Tu), both enhanced Htt secretion (Fig. 2, *C*–*F*). Under these experimental conditions, no cell death was detected by the LDH cytotoxicity assay (Fig. S2*C*). These results confirm that Htt secretion is elevated under stress conditions.

### GRASP55 level is upregulated by cellular stress and Htt expression

**Figure 3:**
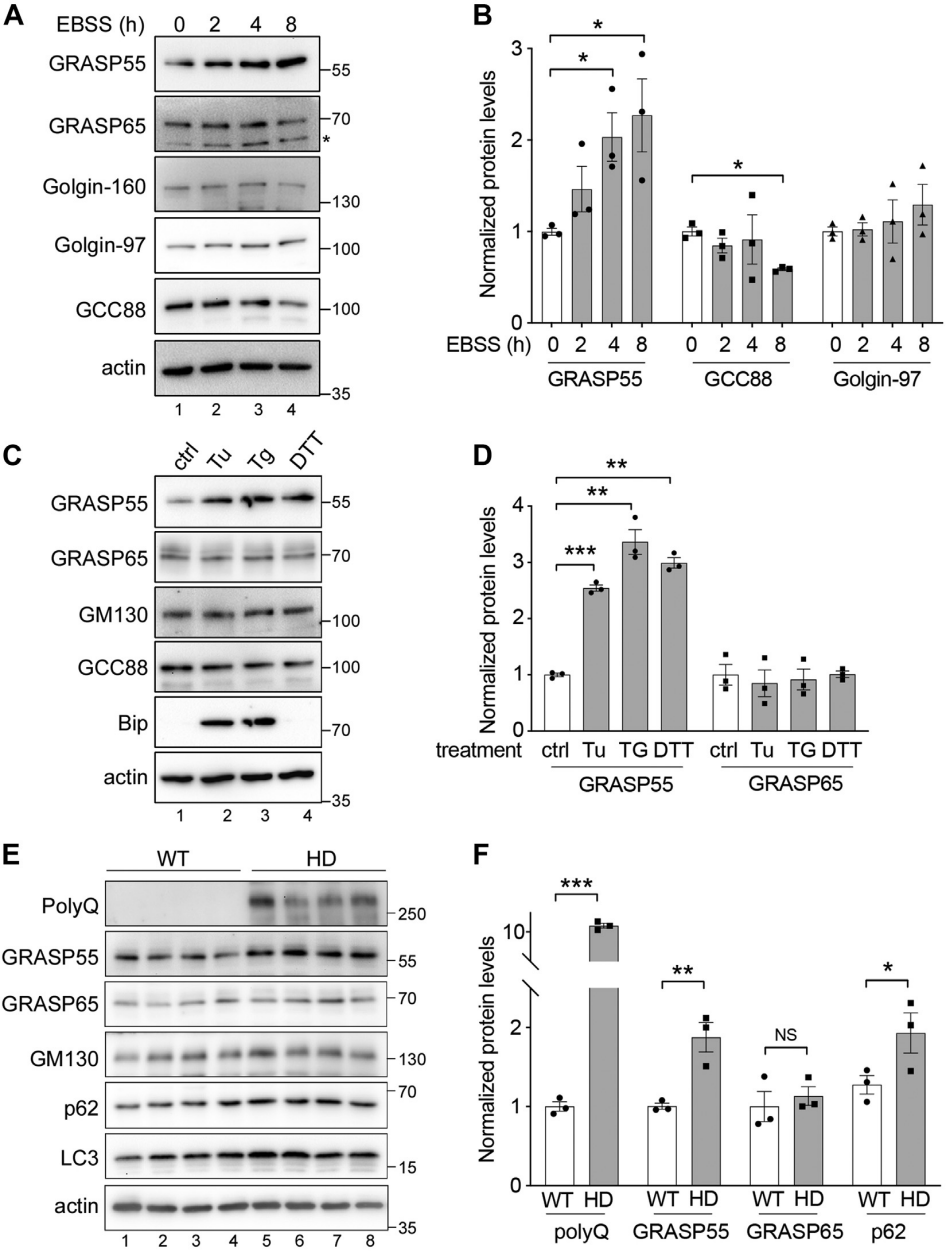
**GRASP55 expression is upregulated under different stress conditions.***A*, GRASP55 level is increased after amino acid starvation. WT HeLa cells were treated with EBSS for 0, 2, 4, or 8 h and blotted for indicated Golgi proteins. Note that only GRASP55 is upregulated over time. ∗nonspecific band. *B*, quantitation of indicated proteins in *A*. *C*, GRASP55 expression is upregulated by ER stress. Cells were treated with 1 μg/μl tunicamycin (Tu), 1 μM thapsigargin (Tg), or 0.5 mM DTT for 24 h and probed for indicated Golgi proteins and an ER stress marker BiP. *D*, quantitation of GRASP55 and GRASP65 in *C*. *E*, GRASP55 expression is elevated in Htt transgenic mice. Brain samples of 8- to 10-month-old Htt transgenic mice (HD; Htt-Q200 expressed under the Htt protomer in knock-in mice) and WT littermates were analyzed by Western blot of indicated proteins. Only GRASP55 increased its level in HD mice compared with WT. *F*, quantitation of indicated proteins in *E*. Results are presented as mean ± SEM; statistical analysis was performed using Student’s *t* test. ∗*p* < 0.05; ∗∗*p* < 0.01; and ∗∗∗*p* < 0.001. BiP, binding protein; EBSS, Earle′s balanced salt solution; ER, endoplasmic reticulum; GRASP, Golgi reassembly stacking protein; Htt, huntingtin.

The fact that GRASP55 localization and function are regulated by cellular stressors suggests that GRASP55 may serve as both a stress sensor and an effector (41). In addition to its relocalization from the Golgi to autophagosomes under starvation conditions (38), we wondered whether GRASP55 level might also be upregulated to meet the high demand for its roles in Golgi structure formation, autophagy, and unconventional secretion in response to stress. To test this hypothesis, we incubated WT HeLa cells in EBSS for 0, 2, 4, and 8 h and measured GRASP55 level by Western blot. Indeed, EBSS treatment increased GRASP55 expression but had no significant effect on other Golgi proteins we tested, including GRASP65, Golgin-160, Golgin-97, and GCC88 (Fig. 3, *A* and *B*). The elevation of GRASP55 expression was not limited to amino acid starvation, as it also applied to ER stress. When cells were treated with ER stress inducers, including Tu, thapsigargin, and DTT, the level of GRASP55 but not that of other Golgi proteins increased (Fig. 3, *C* and *D*).

Since mutant Htt forms aggregates that could induce proteostasis stress, we tested the effect of Htt-Q23 and Htt-Q74 on GRASP55 expression. Both Htt-Q23 and Htt-Q74 expression increased GRASP55 level in cells, although Htt-Q74 exhibited a stronger effect (Fig. S2, *D* and *E*). To validate this observation in mice, we took advantage of a previously established knock-in mouse model that expresses full-length Htt-Q200 in the brain (28, 42). These studies have shown that expression of Htt-Q200 upregulated both LC3-II and p62 levels compared with control, indicating an enhanced autophagosome formation but reduced lysosomal degradation. This conclusion was validated in our Htt mice and cell samples, as indicated by p62 accumulation (Figs. 3, *E* and *F*, S2, *D* and *E*) (42). In response to the proteostasis stress, GRASP55 level was also elevated in the brain tissues, whereas other proteins such as GRASP65 and GM130 did not change (Fig. 3, *E* and *F*). Taken together, GRASP55 level is upregulated by cellular stress and Htt expression, possibly to meet the high demand of unconventional secretion.

### GFP-Htt-Q74 secretion is autophagy dependent

**Figure 4:**
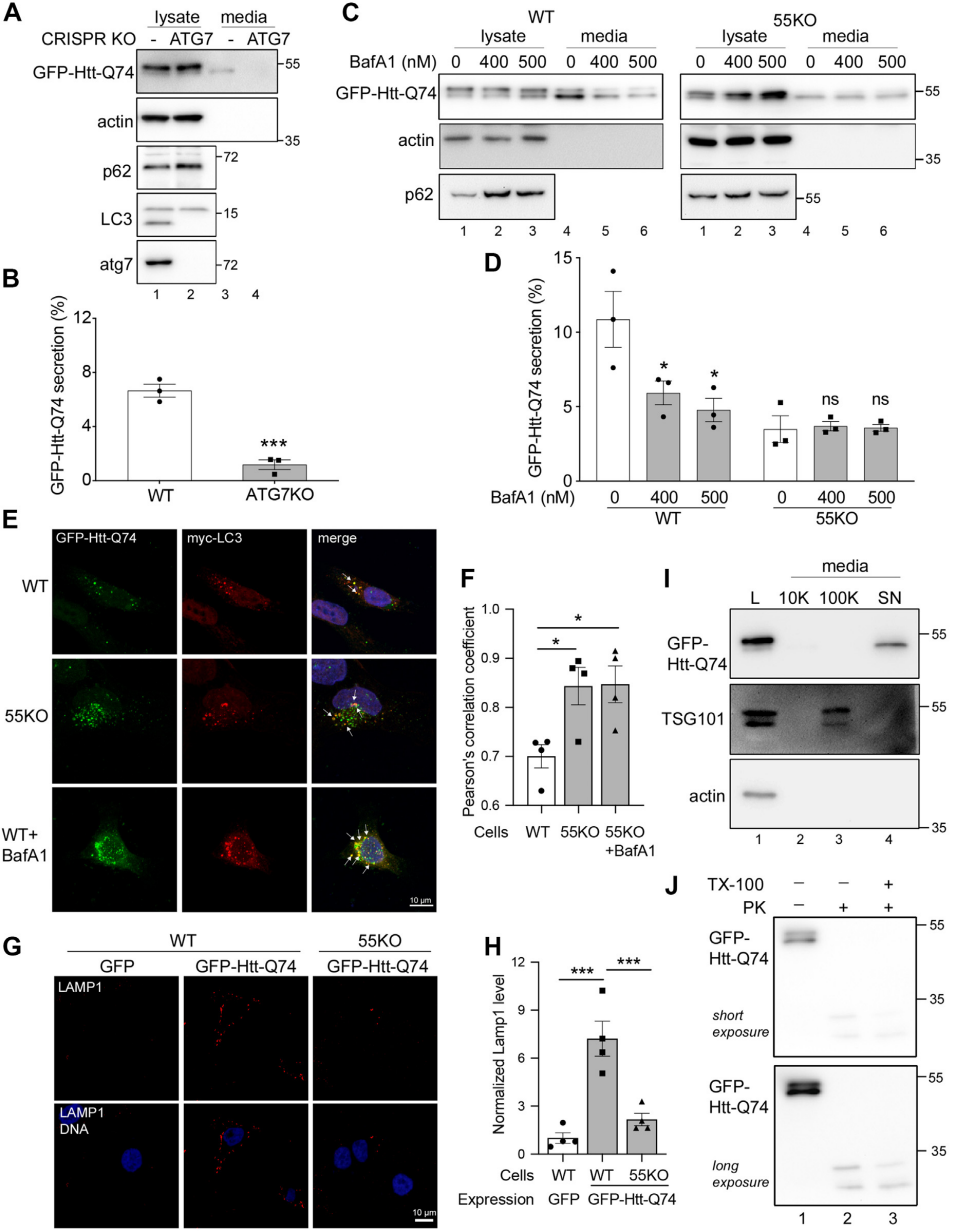
**Htt secretion is autophagy dependent.***A*, Htt secretion is inhibited by ATG7 KO. WT and ATG7 KO MEF cells were transfected with GFP-Htt-Q74 and analyzed for Htt secretion by Western blot. *B*, quantitation of Htt secretion in *A*. *C*, Htt secretion is reduced by autophagy inhibition. WT and 55KO HeLa cells expressing GFP-Htt-Q74 were treated with 0, 400, or 500 nM BafA1 for 4 h and analyzed for Htt secretion by Western blot. Note that BafA1 reduced Htt secretion in WT but not 55KO cells. *D*, quantitation of Htt secretion in *C*. *E*, Htt colocalizes with autophagosomes. Htt-expressing cells were prepermeabilized with 0.1% saponin for 2 min, fixed, and immunostained with a myc antibody. *F*, quantitation of Htt and LC3 colocalization in *E*. *G*, Htt is likely released by lysosome exocytosis. WT HeLa cells expressing GFP or GFP-Htt-Q74 were stained for LAMP1 without permeabilization. *H*, quantitation of cell surface LAMP1 level in *G*, with LAMP1 GFP–expressing WT cells normalized to 1. Results are presented as mean ± SEM; statistical analysis was performed using Student’s *t* test. ∗*p* < 0.05; ∗∗*p* < 0.01; and ∗∗∗*p* < 0.001. *I*, Htt secretion is independent of exosomes. Conditioned medium from GFP-Htt-Q74–expressing cells was harvested and subjected to differential centrifugation. Equal proportions of each pellet and soluble fraction were analyzed by Western blot for actin, TSG101, and GFP. L, whole cell lysate; 10 K, 10,000*g* 30 min pellet; 100 K, 100,000*g* 60 min pellet; SN, 100,000*g* 60 min supernatant. *J*, secreted Htt is not enclosed in membrane structures. Conditioned medium from GFP-Htt-Q74–expressing cells was treated with 2.5 μg/ml proteinase K (PK) for 10 min in the presence or the absence of Triton X-100 followed by Western blot of GFP. BafA1, bafilomycin A1; Htt, huntingtin; 55KO, GRASP55 KO; LAMP, lysosomal membrane–associated protein; MEF, mouse embryonic fibroblast.

The fact that GRASP55 expression, autophagy, and unconventional secretion are all elevated under stress conditions implies that these events are related. To test if Htt secretion depends on autophagy, we expressed GFP-Htt-Q74 in the ATG7 KO mouse embryonic fibroblast (MEF) cell line established by Dr Masaaki Komatsu (43) in which autophagosome formation was abolished, as indicated by the lack of LC3-II (Fig. 4*A*). Htt secretion was reduced in ATG7 KO cells to an almost undetectable level (Fig. 4, *A* and *B*). In addition, inhibition of autophagosome–lysosome fusion by bafilomycin A1 (BafA1) treatment also decreased Htt secretion in WT but not 55KO cells (Fig. 4, *C* and *D*).

The results described previously are consistent with the role of GRASP55 in autophagosome–lysosome fusion, suggesting that GRASP55 regulates Htt unconventional secretion *via* controlling autophagosome maturation. If this conclusion is correct, we would expect to see more colocalization of Htt with the autophagosome marker LC3 in 55KO and BafA1-treated cells. Consistent with this idea, Htt partially colocalized with LC3 in WT cells, whereas 55KO and BafA1 treatment enhanced their colocalization (Fig. 4, *E* and *F*). Taken together, these results demonstrated that Htt is released through an autophagy-dependent mechanism.

### Mutant Htt is released by lysosome exocytosis

It has been indicated that unconventional secretion could occur through lysosomal secretion (15). Indeed, GFP-Htt-Q74 expression, but not GFP expression, enhanced the signal of the lysosome protein LAMP1 at the cell surface, whereas this effect was highly reduced in 55KO cells (Fig. 4, *G* and *H*). Consistent with the notion that GFP-Htt-Q74 is secreted *via* lysosome exocytosis, long exposure of the gels reveals an intermediate form of GFP-Htt-Q74 and free GFP in the cell lysate (Fig. S1*G*), indicating a possibility that some GFP-Htt-Q74 molecules might be partially processed in lysosomes prior to their secretion.

To obtain evidence that mature lysosomal enzymes are also secreted under stress conditions in a similar pattern as Htt, we analyzed cathepsin D secretion in the same Htt secretion assays under different stress conditions, including glucose and amino acid starvation, and inhibition of proteasomal degradation by MG132 treatment. As shown in Figure 2, *A*–*D*, both starvation and proteostatic stress conditions strongly enhanced the secretion of both Htt and mature cathepsin D. We also induced ER stress by Tu treatment, which has been previously reported to preferentially increase the precursor but not mature cathepsin D secretion (44). As shown in Figure 2, *E* and *F*, although the amount of mature cathepsin D in the conditioned media was low, Tu treatment increased the secretion of both mature and proforms of cathepsin D. In addition, we also inhibited lysosome exocytosis by vacuolin-1 treatment (45) and tested its effect on Htt secretion. The result showed that vacuolin-1 treatment reduced amino acid starvation–induced Htt and mature cathepsin D secretion (Fig. S3). These results indicate that Htt is taken up by autophagosomes, and once autophagosomes are fused with lysosomes, Htt can be released by autolysosome/lysosome exocytosis.

It has previously been shown that Htt is secreted in exosomes (11), which is in conflict with our conclusion that Htt secretion is autophagy dependent. Therefore, we performed differential centrifugation to pellet exosomes in the conditioned media and determined the level of Htt in different fractions. As shown in Figure 4*I*, exosomes were highly enriched in the pellet of the 100,000*g* spin as indicated by the exosome marker TSG101 (lane 3), whereas Htt was only found in the supernatant (lane 4). To confirm that Htt was not secreted in membrane-bound structures such as exosomes, we treated the conditioned media of Htt-expressing cells with proteinase K with or without Triton X-100 (TX-100). The results showed that Htt was degraded by proteinase K regardless of whether TX-100 was included (Fig. 4*J*). Taken together, these results demonstrate that Htt secretion depends on autophagy and lysosome exocytosis but not exosome secretion.

### GRASP55 facilitates GFP-Htt-Q74 secretion through promoting autophagosome maturation and stabilizing p23/TMED10

**Figure 5:**
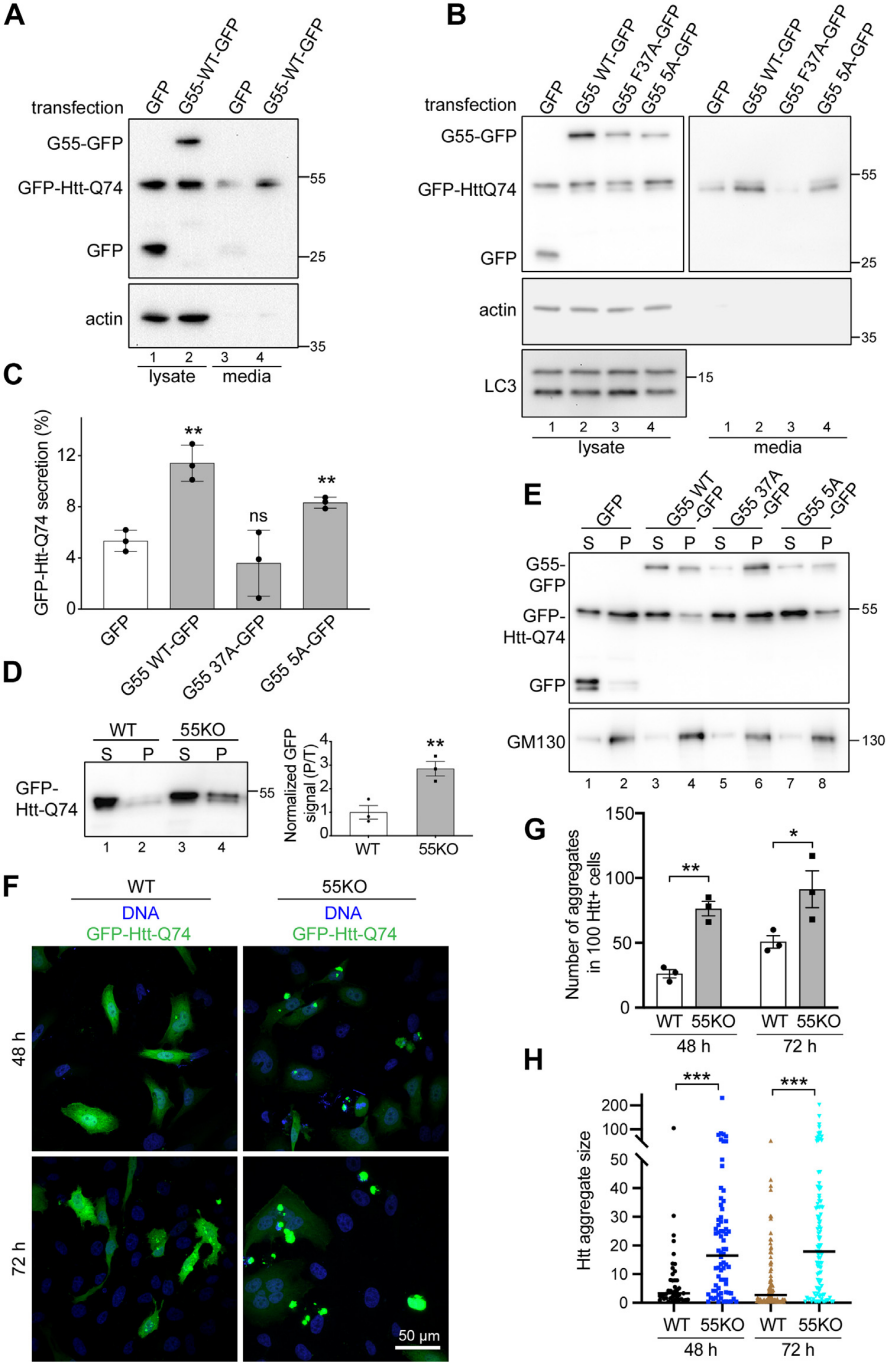
**GRASP55 regulates Htt secretion *via* autophagy and p23.***A*, GRASP55 expression rescues the defect of Htt secretion in 55KO cells. 55KO cells were first transfected with GFP-Htt-Q74 for 24 h and then with either GFP or WT GRASP55-GFP for another 24 h. Htt secretion was analyzed by Western blot. *B*, expression of WT GRASP55, but not its autophagy inactive F37A mutant, rescues the defect of Htt secretion in 55KO cells. GFP-Htt-Q74–expressing 55KO cells were transfected with GFP, WT GRASP55-GFP, or its F37A or 5A mutant for 24 h. Htt secretion was analyzed by Western blot. *C*, quantitation of Htt secretion in *B*. *D*, Htt aggregation is enhanced by 55KO. WT and 55KO cells were transfected by GFP-Htt-Q74 for 48 h and lysed by 0.5% NP-40 followed by centrifugation at 136,000*g* at 4 °C for 1 h. Supernatant (S) and pellet (P) were loaded at a 1:6 ratio and blotted for GFP. T, total. *E*, expression of WT GRASP55 but not F37A reduces Htt aggregation in 55KO cells. 55KO cells transfected as in *B* were analyzed for Htt aggregation as in *D*. *F*, Htt forms more aggregates in 55KO than WT cells. WT and 55KO cells were transfected with GFP-Htt-Q74 for 48 h (*upper panels*) or 72 h (*lower panels*), stained for DNA, and imaged. *G*, quantification of the number of Htt aggregates in *F*. *H*, quantification of the size of Htt aggregates in *F*. Htt aggregate size quantification is performed using NIS-Elements software simple region of interest (ROI) editor function. Results are presented as mean ± SEM; statistical analysis was performed using Student’s *t* test. ∗*p* < 0.05; ∗∗*p* < 0.01; and ∗∗∗*p* < 0.001. GRASP, Golgi reassembly stacking protein; Htt, huntingtin; 55KO, GRASP55 KO.

GRASP55 facilitates autophagosome maturation by tethering autophagosomes and lysosomes through the interactions with LC3-II on autophagosomes and LAMP2B on lysosomes (38). To confirm that GRASP55 promotes Htt secretion through regulating autophagosome maturation, we re-expressed GRASP55 and its autophagy mutants in 55KO cells and tested their effect on Htt secretion. Expression of WT GRASP55 restored GFP-Htt-Q74 secretion, showing that the reduced Htt secretion in 55KO cells was specific for GRASP55 depletion (Fig. 5*A*). In contrast, expression of the F37A mutant of GRASP55, which abolishes GRASP55–LC3 interaction (38), failed to rescue the secretion defect of Htt in 55KO cells (Fig. 5, *B* and *C*). The 5A mutant of GRASP55, whose O-GlcNAcylation is disabled and thus its role in autophagy is constitutively active, also rescued Htt secretion similar to WT GRASP55. In this experiment, GRASP55 itself was not secreted with Htt; and we did not detect an interaction between Htt and GRASP55 by coimmunoprecipitation. These results demonstrate that GRASP55 facilitates Htt secretion by promoting autophagosome maturation.

Recently, it has been reported that an ERGIC protein p23, also known as TMED10, functions as a protein channel for cytosolic proteins to be translocated into the ERGIC lumen before they are transported to autophagosomes and secreted to the extracellular space (46). Indeed, p23 depletion largely reduced GFP-Htt-Q74 secretion (Fig. S4*A*). Considering that some members of the p24 family proteins interact with GRASP65 (47) and that GRASP55 is partially relocated to the ER under stress conditions for CFTR trafficking (29), inhibition of Htt secretion by p23 depletion and 55KO suggested that GRASP55 may function together with p23 in Htt secretion. This speculation was first supported by the observation that the p23 level was reduced in 55KO but not 65KO cells (Fig. S4, *B* and *C*). In addition, p23 coimmunoprecipitated with GRASP55 but not GRASP65, whereas known interactions between GRASP65 and GM130 and between GRASP55 and Golgin-45 were readily detectable in the same experiment (Fig. S4*D*). These results support the idea that GRASP55 interacts with p23 to facilitate Htt unconventional secretion.

### Inhibition of Htt secretion by 55KO enhances Htt aggregation and toxicity in the cell

It is generally believed that HD is caused by the accumulation of toxic mutant Htt protein in the cell. To determine the functional significance of Htt secretion, we assessed Htt aggregation in WT and 55KO cells. We lysed GFP-Htt-Q74–expressing cells in detergent and separated Htt aggregates from its soluble form by centrifugation. Western blot analysis of the two fractions showed that Htt formed more aggregates in 55KO than WT cells (Fig. 5*D*). Expression of WT GRASP55 and its 5A mutant, but not the autophagy-inactive F37A mutant, reduced Htt aggregation in 55KO cells (Fig. 5*E*), consistent with their roles in Htt secretion. In this experiment, a significant amount of GRASP55 and GM130 was found in the pellet (Fig. 5*E*), consistent with the idea that they form a detergent-insoluble Golgi matrix (48, 49). We also confirmed the effect of GRASP55 KO on Htt aggregation by microscopy. At both 48 and 72 h post-transfection, 55KO cells showed more and larger aggregates compared with WT (Fig. 5, *F*–*H*). These results demonstrate that GRASP55-dependent Htt secretion reduces its aggregation and toxicity in the cell.

As Htt aggregation causes neurodegeneration, we tested Htt secretion in neuronal cells. First, in Neuro2A (N2A) neuronal cells, transfected GFP-Htt-Q74 was detected in the conditioned medium, and its level was reduced by GRASP55 depletion (Fig. S5*A*). Similar to HeLa cells, GRASP55 depletion also increased GFP-Htt-Q74 aggregation in N2A cells (Fig. S5*B*). Second, to determine if full-length Htt-polyQ stably expressed under the endogenous promoter is also secreted in a GRASP55-dependent manner, we assessed Htt-polyQ secretion using the striatal-derived cell line ST*Hdh*Q111/Q111 (50). Indeed, full-length Htt was secreted as previously reported (11), and more importantly, its secretion was inhibited by GRASP depletion (Fig. S5*C*). Furthermore, GRASP55 depletion also enhances the aggregation of full-length Htt-polyQ in striatal cells (Fig. S5*D*). These results validated our finding that GRASP55 facilitates mutant Htt secretion in neuronal cells.

### Secretome analysis of WT and 55KO cells identifies new proteins secreted by GRASP55-dependent unconventional secretion

**Figure 6:**
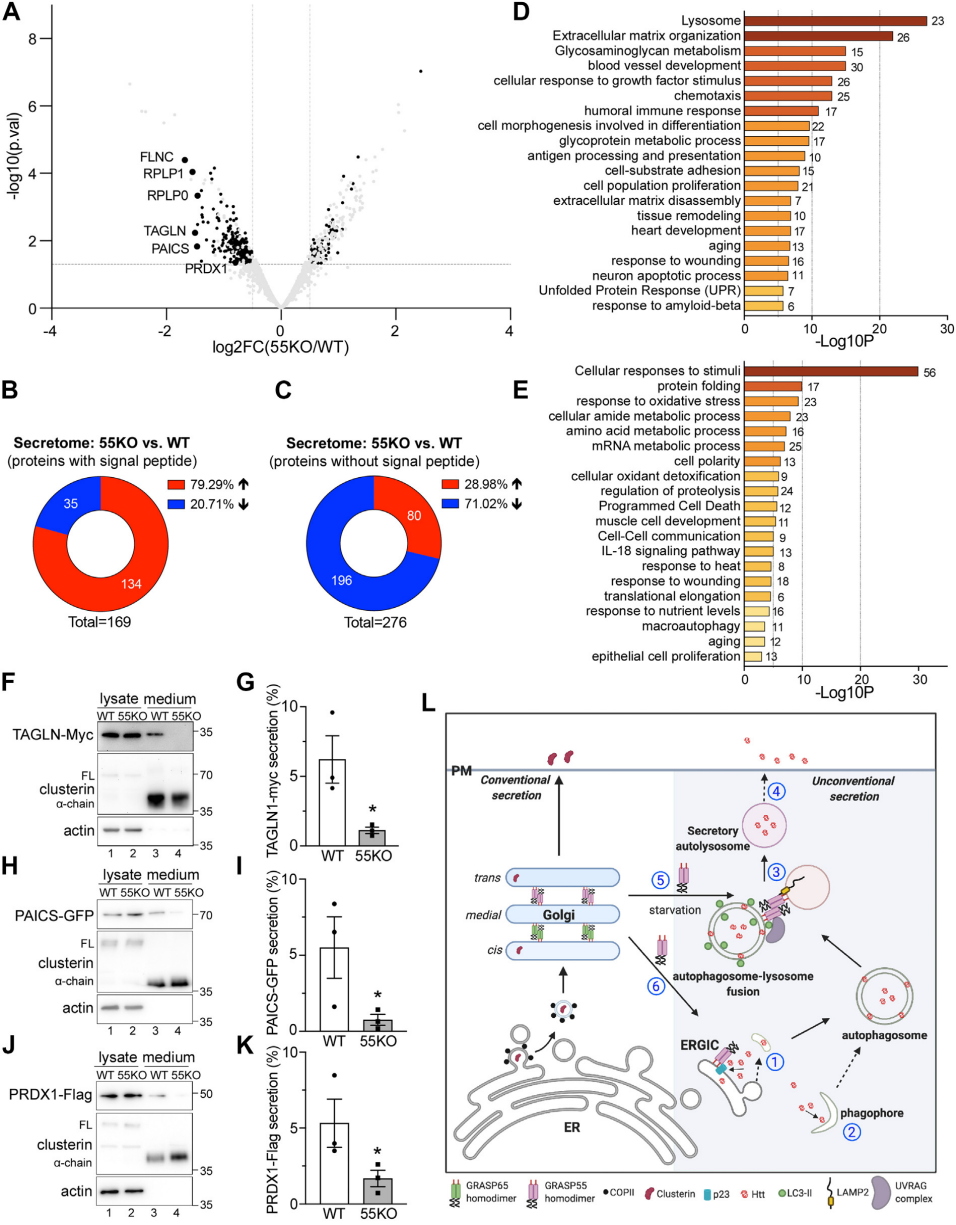
**Secretome analysis identifies candidate proteins secreted by GRASP55-dependent unconventional secretion.***A*, volcano plot of candidate proteins in 55KO *versus* WT secretome. *Black dots* represent significantly altered cargoes without signal peptide. All other cargoes are shown in *gray*. A few top candidates (*large black dots*) in the unconventional pathway are labeled. *B*, GRASP55 KO increases overall conventional secretion. Indicated are the numbers of candidate proteins with ER signal sequences whose secretion was significantly increased or decreased in 55KO compared with WT cells (|Log2FC [55KO/WT]|>0.5; *p* < 0.05). *C*, GRASP55 KO decreases overall unconventional secretion. Number and percentage of candidates with no signal sequence that are increased or reduced in GRASP55 KO secretome compared with WT. *D*, Metascape Gene Ontology (GO) enrichment analysis of identified candidate proteins with predicted ER signal sequences whose secretion was significantly altered in 55KO compared with WT. The number of proteins in each pathway is indicated on the *right of the graph*. *E*, GO enrichment analysis of candidate proteins without predicted ER signal sequences whose secretion was significantly altered. *F*–*K*, TAGLN (transgelin 1), PAICS (multifunctional protein ADE2), and PRDX1 (peroxiredoxin-1) are secreted in a GRASP55-dependent manner. Western blot and quantitation of each protein are shown. Results are presented as mean ± SEM; statistical analysis was performed using Student’s *t* test. *L*, GRASP55 functions in conventional and unconventional protein secretion. In the conventional secretory pathway (*left*), secretory proteins such as clusterin are transported from the ER to the Golgi and then to the plasma membrane (PM). GRASP55 depletion–mediated Golgi unstacking enhances the overall secretion of cargoes in the conventional pathway, as demonstrated for Figure 6*B*. In the unconventional pathway induced by stress (*right*), cytosolic proteins such as Htt may enter autophagosomes either *via* p23/TMED10-mediated translocation into the ERGIC lumen (1), from where it is incorporated into autophagosomes, or by direct sequestration into phagophores (2). Htt in autophagosomes is then transferred to autolysosomes by membrane fusion (3) and released by lysosome exocytosis (4). Under stress conditions such as energy deprivation, GRASP55 is relocated from the Golgi to the autophagosome and lysosome interface to facilitate their fusion (5) or to the ERGIC to interact with p23/TEMD10 and regulate cytosolic protein translocation into the lumen (6). Depletion of both p23 and GRASP55 reduced Htt secretion, indicating that the dominant pathway for Htt translocation and secretion is (1) and (4). ER, endoplasmic reticulum; ERGIC, ER–Golgi intermediate compartment; GRASP, Golgi reassembly stacking protein; 55KO, GRASP55 KO.

To identify native proteins that are secreted in a GRASP55-dependent mechanism, we analyzed the conditioned media of WT and 55KO cells by tandem mass tag (TMT) labeling and LC–MS. With a 1% false discovery rate, we identified 1696 proteins in the media (Table S1). We plotted all 1696 secreted proteins on a volcano plot to display their changes in the 55KO *versus* WT secretome (Fig. 6*A*). Significantly changed cargoes (|Log2FC [55KO/WT]|>0.5, *p* < 0.05) without predicted signal peptides are shown in *black*. On this plot, a majority of the unconventional targets were reduced in 55KO.

Next, we systematically analyzed the impact of GRASP55 depletion on the secretion of both conventional and unconventional pathway cargoes. Among the 1696 proteins, we identified a total of 445 proteins whose secretion was highly affected by 55KO (|Log2FC [55KO/WT]|>0.5, *p* < 0.05) (Table S1). Within these 445 significantly changed proteins, 169 contained an ER signal sequence predicted by the SignalP-5.0 server (http://www.cbs.dtu.dk/services/SignalP), whereas 276 proteins did not appear to have ER signal peptides (Table S1). Within the 169 proteins with ER signal sequences, 134 (79%) of them were increased in 55KO secretion (Fig. 6*B*), consistent with our previous findings that GRASP depletion accelerates protein trafficking (51). In contrast to the proteins with an ER signal sequence, 196 (71%) of the 276 proteins without ER signal sequences were reduced in 55KO secretion (Fig. 6*C*). It is worth mentioning that a GRASP55-dependent secretome has recently been reported (52). But unlike this report in which all proteins, including those with (*e.g.*, matrix metalloproteinase-2 [MMP2]) and without signal peptides, are included in unconventional secretion, we further differentiated the proteins into two separate groups and showed different effects by 55KO. Taken together, these results support our conclusion that GRASP55 plays essential roles in unconventional protein secretion.

To identify the pathways affected by 55KO, we performed Gene Ontology (GO) analysis of the significantly changed proteins. GO term pathway analysis of the 169 proteins with predicted ER signal peptides identified the following pathways as significantly changed in 55KO: lysosomal enzymes and structural proteins, extracellular matrix organization, glycosaminoglycan metabolism, cell adhesion and proliferation, cellular response to growth factor stimuli, immune response and tissue remodeling, and response to amyloid-beta, unfolded protein response, and aging (Fig. 6*D* and Table S2). The most affected pathway is related to lysosomal biogenesis, consistent with our previous finding that Golgi unstacking by GRASP depletion causes missorting and increased secretion of lysosomal enzymes (51). Given the central role of the Golgi in protein trafficking, glycosylation, and secretion, it is also reasonable to see that GRASP55 depletion significantly alters extracellular matrix organization and glycosaminoglycan metabolism (53). In addition, our secretome analysis also validated our previous findings that 55KO affects cell adhesion, migration, and growth (54).

GO term analysis of the 276 significantly changed proteins without signal sequences identified pathways such as cellular response to stimuli, oxidative stress, nutrient levels, heat, wounding, protein folding, macroautophagy, programmed cell death, and cell–cell communication (Fig. 6*E* and Table S2), which are consistent with the role of GRASP55 in stress response (41). These findings further suggest a link between GRASP55, autophagy-dependent unconventional secretion, and stress response. Consistent with this notion, previously identified unconventional secretion cargoes such as SOD1 and interleukin-β1 are related to oxidative stress and inflammation, and their secretion is increased under certain stress conditions such as nutrient starvation (24, 55).

To confirm that the identified proteins without ER signal sequences are indeed secreted through a GRASP55-dependent mechanism, we tested a few top candidates (Fig. 6*A*), including transgelin 1 (TAGLN), multifunctional protein ADE2 (PAICS), and peroxiredoxin-1 (PRDX1), using the secretion assay we established for Htt. These proteins were selected because they are predicted to be highly secreted by the SecretomeP 2.0 server (http://www.cbs.dtu.dk/services/SecretomeP), an online tool to predict the probability of unconventional protein secretion, although their secretion and the GRASP55 dependence in the secretion have not been experimentally confirmed previously. Indeed, these proteins were all detected in the conditioned media, and their secretion was all reduced in 55KO cells (Fig. 6, *F*–*K*). In summary, the unbiased secretomic analysis provided new evidence that GRASP55 depletion accelerates conventional protein trafficking and secretion but inhibits unconventional secretion. It also identified a list of *bona fide* candidates that are secreted by GRASP55-dependent unconventional secretion.

## Discussion

In this study, we used GFP-Htt-Q74 as a model protein to study the role of GRASP55 in unconventional secretion and cytosolic aggregation of neurotoxic proteins. We discovered that mutant Htt is secreted through a GRASP55- and autophagy-dependent but Golgi-independent pathway. Our results also revealed a link between enhanced Htt secretion, elevated autophagy, and increased GRASP55 expression under various stress conditions and confirmed a novel role of GRASP55 in stress response (41). Htt may enter autophagosomes through p23-mediated translocation or *via* direct sequestration by phagophores. Because of lysosomal dysfunction, undegraded proteins in autolysosomes and lysosomes could be released by lysosome exocytosis (Fig. 6*L*). GRASP55 enhances Htt secretion through two actions, one is to facilitate autophagosome–lysosome fusion by interacting with LC3 and LAMP2, and another is to stabilize p23, the translocon that allows cytosolic proteins to enter the lumen of the ERGIC, from where they are transported to autophagosomes. Finally, using systematic secretomic analysis, we identified and cataloged new proteins in GRASP55-dependent unconventional secretion.

It is interesting to see that GRASP55-dependent unconventional secretion is selective, at least to some degree. First, among all marker proteins tested, Htt and SOD1 are preferably secreted than TDP-43 and tau. Consistently, the secretion of Htt and SOD1, but not TDP-43 and tau, depends on GRASP55. It has been previously shown that tau is secreted *via* direct translocation across the plasma membrane, which is regulated by heparan sulfate and *PtdIns*(4,5)P2 (56). So far, there is no indication that GRASP55 is involved in this pathway, although GRASP55 depletion increases heparan sulfate synthesis but decreases its sulfation and secretion (53). TDP-43, on the other hand, has been shown to be secreted in a prion-like manner *via* exosomes (57). While it is not clear whether GRASP55 directly regulates exosome secretion, our data indicate that it plays no role in TDP-43 secretion. Second, Htt-Q74 is preferentially secreted than Htt-Q23. Given that Htt-Q74 forms more aggregates than Htt-Q23, one possible explanation is that aggregated proteins are selectively secreted. This speculation is supported by the third observation that of the two bands of Htt-Q74, only the aggregation-prone dephosphorylated form is secreted. In addition, Q80-GFP alone is secreted to a similar level as Htt-Q74. Whether Htt-Q74 aggregates are preferably enclosed by autophagosomes or selectively translocated into the ERGIC lumen requires further investigation.

In HD, mutant Htt with a long polyQ stretch tends to form toxic protein aggregates that cause cellular toxicity and neurodegeneration (3, 6, 58). In this study, we used the N-terminal fragment of Htt as a cargo because of its relevance to the disease. Exon 1 fragment of Htt contains the polyQ stretch, which is the source of the toxicity of mutant Htt. This fragment is harder to degrade, more toxic than full-length Htt, and detected in the CSF with an elevated concentration in HD (25, 26). Like full-length Htt, the aggregation and toxicity of exon 1 fragment are regulated by phosphorylation of S13 and S16, which indicates similar regulation. For these reasons, we believe that the N-terminal fragment of Htt serves as an excellent reporter in our study, and we have also confirmed our key findings with full-length Htt.

Autophagy is an energy and nutrient deprivation–induced lysosome-mediated degradation pathway essential for organelle turnover and degradation of aggregated proteins. Compared with non-neuronal cells, neurons need to maintain high basal autophagy for survival (59, 60). Autophagy and autophagy defects have been linked to neurodegenerative diseases (34, 61). While WT Htt is required for selective autophagy (62), mutant Htt has been shown to hinder autophagy flux, and a certain population of toxic Htt is immune to autophagy-dependent degradation (63). It is possible that defects in lysosomal function and impairment of protein degradation may activate GRASP55-dependent unconventional secretion to clear out the toxic degradation-resistant Htt aggregates. Indeed, our data demonstrate that inhibition of proteasomal degradation pathway also significantly increases unconventional secretion of mutant Htt.

Htt secretion could be a double-edged sword to the neuronal cells and the disease. On the one hand, the aggregation-prone dephosphorylated Htt with a long polyQ stretch is selectively secreted, which reduces the amount of Htt aggregates in the cells and subsequently ameliorates its toxicity in the cell. In this sense, Htt secretion is beneficial to the cell. Our results support this hypothesis as GRASP55 depletion increases overall Htt aggregation in the cell (Figs. 5, *D*–*H* and S5). On the other hand, secreted Htt aggregates may serve as seeds for internalization into the neighboring cells to trigger more Htt aggregation. Here, Htt secretion may facilitate the spreading and propagation of Htt aggregates from disease to healthy neurons, which may contribute to the development of the disease. Future studies will test the biological significance of Htt secretion through the intervention of GRASP55 in HD animal models. New information provided in this study may shed light on novel therapeutic approaches for the treatment of this devastating disease.

## Experimental procedures

### Reagents, plasmids, and antibodies

All reagents were purchased from Sigma–Aldrich, Roche, Calbiochem, and Fisher unless otherwise stated. The following complementary DNA constructs have been described previously: pEGFP-N1-GRASP55 (WT, 37A, 5A), pEGFP-N1-GRASP65 (WT), and pEGFP-N1-Q80 (38, 64). Other complementary DNAs include GFP-Htt-Q74 (Addgene; catalog no.: 40262), GFP-Htt-Q23 (Addgene; catalog no.: 40261), TAGLN-Myc (OriGene; catalog no.: RC215789), PAICS-EGFP (Addgene; catalog no.: 99108), and pFRT/TO/HIS/FLAG/HA-PRDX1 (Addgene; catalog no.: 38086).

Antibodies used in this study include monoclonal antibodies against β-actin (Proteintech; catalog no.: 66009-1-Ig), clusterin (Santa Cruz; catalog no.: SC-5289), FLAG (Sigma; catalog no.: M1804), GFP (Proteintech; catalog no.: 66002-1-lg), Myc (9E10; Dr David Sheff), and PolyQ (Sigma; catalog no.: MAB1574); polyclonal antibodies against Atg7 (Cell Signaling; catalog no.: 2631S), BiP (binding protein; Santa Cruz; catalog no.: sc-13968), cathepsin D (Cell Signaling; catalog no.: 69854), GCC88 (Proteintech; catalog no.: 16271), GCK (Proteintech; catalog no.: 19666-1-AP), Golgin-97 (Proteintech; catalog no.: 12640-1-AP), Golgin-160 (Proteintech; catalog no.: 21193-1-AP), GRASP55 (Proteintech; catalog no.: 10598-1-AP), human GRASP65 (Dr Joachim Seemann, University of Texas Southwestern Medical Center), HK1 (Proteintech; catalog no.: 19662-1-AP), α5 integrin (Cell Signaling; catalog no.: 4705), LC3 (Cell Signaling; catalog no.: 2755), and p62 (Proteintech; catalog no.: 18420-1-AP).

### Cell culture, transfection, and treatment

For cell culture, HeLa, N2A, and MEF cells were grown in DMEM (Invitrogen) containing 10% super calf serum (Gemini; catalog no.: 100-510) and glutamax at 37 °C in a 5% CO_2_ incubator. ST*Hdh*Q111/Q111 cells (Coriell Institute for Medical Research) were cultured in the same medium with the addition of 400 μg/ml G418. For Htt expression, GFP-Htt-Q74 was transfected into WT or GRASP KO HeLa cells in 3.5 cm dishes when the cells reached the confluency of 50 to 60%. For drug treatment, 5 μg/ml BFA, 500 ng/ml nocodazole, 100 μg/ml cycloheximide, 100 μM MG132, 5 μM vacuolin-1, 1 μM STS, 1 μM OA, or 5 μg/ml Tu was directly applied to the medium for the indicated times.

### Protein secretion assay and trichloroacetic acid precipitation

WT or GRASP KO HeLa cells grown in 3.5 cm dishes were transfected with GFP-Htt-Q74 (4 μg DNA for a 3.5 cm dish) when the cells reached the confluency of 50 to 60% and incubated in full growth medium for 24 h. Cells were washed with PBS five times and further incubated in 1 ml DMEM without serum for 4 h. Conditioned media were collected and cleared by centrifugation at 500*g* for 10 min at 4 °C and 2000*g* for 20 min at 4 °C. About 0.9 ml of precleared conditioned medium was then mixed with 0.11 volumes of ice-cold 100% trichloroacetic acid (TCA; 2.2 g/ml) and placed on ice. After 10 min, 500 μl of ice-cold 10% TCA were added to the sample–TCA mix. After 20 min, samples were centrifuged at 20,000*g* for 30 min at 4 °C. Pellet was washed with cold acetone (−20 °C), dried, and dissolved in 1× SDS loading buffer for Western blot analysis. The cells were washed with cold PBS twice, scraped, and lysed in 20 mM Tris–HCl, pH 8.0, 150 mM NaCl, 1% TX-100, and protease inhibitor cocktail (Thermo) for 30 min on ice. Lysates were cleared by centrifugation (20,000*g* for 20 min at 4 °C). The cell lysate and medium precipitate were loaded at 1:8 ratio. After electrophoresis and transfer, nitrocellulose membranes were incubated with corresponding primary antibodies overnight at 4 °C. The membranes were extensively washed and further incubated with horseradish peroxidase–conjugated goat anti-rabbit or goat antimouse secondary antibodies for 1 h at room temperature and exposed to a FluorChem M machine (Proteinsimple).

To quantify the efficiency of protein secretion, the intensity of the bands of the cargo protein in the conditioned medium and cell lysate on the same gels were quantified using ImageJ (National Institutes of Health), and the percentage of protein secretion was calculated as signal in the conditioned medium/(signal in the conditioned medium + signal in the cell lysate) × 100%, with 1:8 lysate to medium protein loading ratio applied in the equation.

### Immunofluorescence microscopy

Cells were grown on sterile glass coverslips and rinsed with PBS before fixation. To stain total cellular proteins, cells were fixed in 4% paraformaldehyde (PFA) for 10 min and permeabilized with 0.2% TX-100 in PBS for 10 min. Cells were incubated with primary antibodies overnight at 4 °C, washed, and probed with the appropriate secondary antibodies conjugated to tetramethylrhodamine (for 45 min). To stain cell surface LAMP1, cells were fixed with 1% PFA for 10 min without permeabilization. Cells were incubated with an anti-LAMP1 antibody (H4A3; DSHB, 1:100 dilution) overnight at 4 °C, washed, and probed with the appropriate secondary antibodies conjugated to tetramethylrhodamine for 45 min at room temperature. To analyze LC3 and Htt colocalization, cells were prepermeabilized in 125 mM potassium acetate, 25 mM Hepes, pH 7.2, and 2.5 mM magnesium acetate buffer containing 0.1% saponin (prefiltered) for 2 min on ice to release the cytosol and washed by 125 mM potassium acetate, 25 mM Hepes, pH 7.2, and 2.5 mM magnesium acetate buffer for 5 min at room temperature. Cells were then fixed with 4% PFA, permeabilized in 0.2% TX-100, and incubated with primary and secondary antibodies as described previously. DNA was stained with Hoechst 33342 (Thermo Fisher Scientific) in PBS for 5 min. Coverslips were rinsed with PBS and mounted with Mowiol onto slides. Images were taken with a 20× air objective or a 63× oil objective on a Nikon ECLIPSE Ti2 confocal microscope and shown as max projections.

### Protease K protection assay

Protease K protection assay of the conditioned media was performed as previously described (39). Briefly, conditioned medium from HeLa cells expressing GFP-Htt-Q74 was collected as described previously and equally divided into three tubes, one was left untreated on ice, the second was incubated with 2.5 μg/ml Protease K (Thermo Fisher Scientific; catalog no.: AM2542) on ice, and the third was treated with both protease K and 1% TX-100 (from 20% stock) for 10 min on ice. Protease K was then inhibited by adding 1 mM PMSF (from 100 mM stock in isopropanol) and further incubated on ice for 10 min. Proteins in each sample were pelleted by TCA precipitation as aforementioned. The pellets were dissolved in 1× SDS sample buffer (50 mM Tris–HCl, pH 6.8, and 2% SDS) and analyzed by Western blotting.

### Separation of insoluble Htt aggregates from its soluble forms

WT or GRASP KO cells were transfected with GFP-HttQ74 for indicated times. Cells were washed three times with cold PBS, scraped in cold PBS, centrifuged at 1000*g* for 3 min at 4 °C, and lysed in lysis buffer (20 mM Tris 8.0, 150 mM NaCl, 0.5% NP-40, and protease inhibitors) for 20 min on ice. The supernatant and pellet were collected after centrifugation at 55,000 rpm (186,000*g*) for 1 h in a TLA55 rotor. The supernatant concentration was adjusted to 1.5 mg/ml (the volumes of the samples are different) and supplemented with 1/5 volume of 6× SDS loading buffer with DTT (final ×1× SDS in the sample). The pellet was dissolved in the same amount of 2× SDS loading buffer with DTT. Both samples were boiled, and the pellet and supernatant were loaded onto the gel at a 6:1 ratio.

### LDH cytotoxicity assay

LDH cytotoxicity assay was performed following the manufacturer’s instruction (Thermo; catalog no.: 88953). Briefly, 50 μl of conditioned medium was mixed with 50 μl of 1× reaction mixture and incubated in dark at room temperature for 30 min. Then 50 μl of stop solution was added, and the absorbance at 490 and 680 nm (background) was measured. The percentage of LDH cytotoxicity was calculated by dividing the LDH activity of the conditioned medium by the maximal LDH activity of the cell lysate as the kit protocol suggested.

### Proteomics analysis

WT and 55KO cells were cultured in full growth medium in 15 cm dishes in triplicates till they reach 80% confluency. The medium was exchanged to 20 ml DMEM only (without serum) for 12 h; cells and conditioned media were collected. The media were cleaned by centrifugation at 500*g* for 10 min at 4 °C followed by 4000*g* for 15 min at 4 °C, and filtered with a 0.45 μm filter. The media then were concentrated with a 3 kDa cutoff ultrafilter (Millipore; catalog no.: UFC900324), and the protein concentration was determined with Bradford assay (Bio-Rad; catalog no.: 5000006). Cells were lysed in Pierce radioimmunoprecipitation buffer (Thermo; catalog no.: 89900) with a protease inhibitor cocktail, and the protein concentration was tested with Bradford assay. About 75 μg of each sample was provided to the Mass Spectrometry–Based Proteomics Resource Facility at Department of Pathology, University of Michigan for TMT labeling, LC–MS/MS, and bioinformatics analysis.

For TMT labeling, cell and medium samples were proteolyzed and labeled with TMT 10-plex essentially by following manufacturer’s protocol (Thermo Fisher Scientific; catalog no.: 90110; Lot no.: VJ306782). For LC–MS analysis, as previously described (65). An Orbitrap Fusion (Thermo Fisher Scientific) and RSLC Ultimate 3000 nano-UPLC (Dionex) was used to acquire the data. About 2 μl of the sample was resolved on a PepMap RSLC C18 column (75 μm i.d. × 50 cm; Thermo Fisher Scientific) at a flow rate of 300 nl/min using 0.1% formic acid/acetonitrile gradient system (2–22% acetonitrile in 150 min; 22–32% acetonitrile in 40 min; 20 min wash at 90% followed by 50 min re-equilibration), and directly sprayed onto the mass spectrometer using EasySpray source (Thermo Fisher Scientific). Mass spectrometer was set to collect one MS1 scan (Orbitrap; 120 K resolution; automatic gain control [AGC] target 2 × 10^5^; maximum injection time [IT] = 100 ms) followed by data-dependent “Top Speed” (3 s) MS2 scans (collision-induced dissociation; ion trap; normalized collision energy of 35; AGC 5 × 10^3^; and maximum IT of 100 ms). For multinotch-MS3, top 10 precursors from each MS2 were fragmented by higher energy collisional disociation followed by Orbitrap analysis (normalized collision energy of 55; 60 K resolution; AGC 5 × 104; maximum IT of 120 ms, and scan range of 100–500 *m/z*).

Data analysis was performed using Proteome Discoverer (v2.4; Thermo Fisher Scientific). In data analysis, MS2 spectra were searched against SwissProt human protein database (20,353 entries; downloaded on June 20, 2019) with the search parameters: MS1 and MS2 tolerance were set to 10 ppm and 0.6 Da, respectively; carbamidomethylation of cysteines (57.02146 Da) and TMT labeling of lysine and N termini of peptides (229.16293 Da) were considered static modifications; oxidation of methionine (15.9949 Da) and deamidation of asparagine and glutamine (0.98401 Da) were considered variable. Identified proteins and peptides were filtered to retain only those that passed ≤1% false discovery rate threshold. Quantitation was performed using high-quality MS3 spectra (average signal-to-noise ratio of 10 and <50% isolation interference).

### Quantification and statistics

In all figures, the quantification results are expressed as the mean ± SEM from three independent experiments, unless otherwise stated. The statistical significance of the results was assessed using a two-tailed Student’s *t* test. ∗*p* < 0.05, ∗∗*p* < 0.01, and ∗∗∗*p* < 0.001.

### Figure assembly

All figures were assembled using Adobe Illustrator (Adobe, Inc); Figure 6*L* was drawn with BioRender (BioRender.com).

## Data availability

The data that support the findings of this study are available from the corresponding author upon reasonable request.

## Supporting information

This article contains supporting information. It includes four supplemental figures and two supplemental tables. Fig. S1 demonstrates that GRASP55 is required for the secretion of a subset of neurotoxic proteins in addition to Htt. Fig. S2 shows that Q80-GFP is secreted via a GRASP55-dependent mechanism. Fig. S3 shows that inhibition of lysosome exocytosis by vacuolin-1 reduces Htt and mature cathepsin D secretion. Fig. S4 provides evidence that GRASP55 facilitates Htt secretion by stabilizing p23. Fig. S5 shows that GRASP55 controls the secretion and aggregation of full-length Htt-Q111 in striatal cells. Table S1 lists the proteins whose secretion is altered by 55KO in the secretome study. Table S2 lists the selected GO term analysis for the secretome of WT and 55KO cells. This article contains supporting information.

## Conflict of interest

The authors declare that they have no conflicts of interest with the contents of this article.
