# Supplementary material for: GRASP55 regulates the unconventional secretion and aggregation of mutant huntingtin
Source: J Biol Chem. 2022 Jul 1;298(8):102219. doi: 10.1016/j.jbc.2022.102219 (PMC9352920; doi:10.1016/j.jbc.2022.102219)
Supplement: Supporting Information [file mmc3.pdf]

GRASP55 regulates mutant huntingtin unconventional secretion and aggregation

Erpan Ahat, Sarah Bui, Jianchao Zhang, Felipe da Veiga Leprevost, Lisa Sharkey, Whitney Reid, Alexey I. Nesvizhskii, Henry L. Paulson, Yanzhuang Wang

Supporting information

Supplemental Figures and Figure Legends

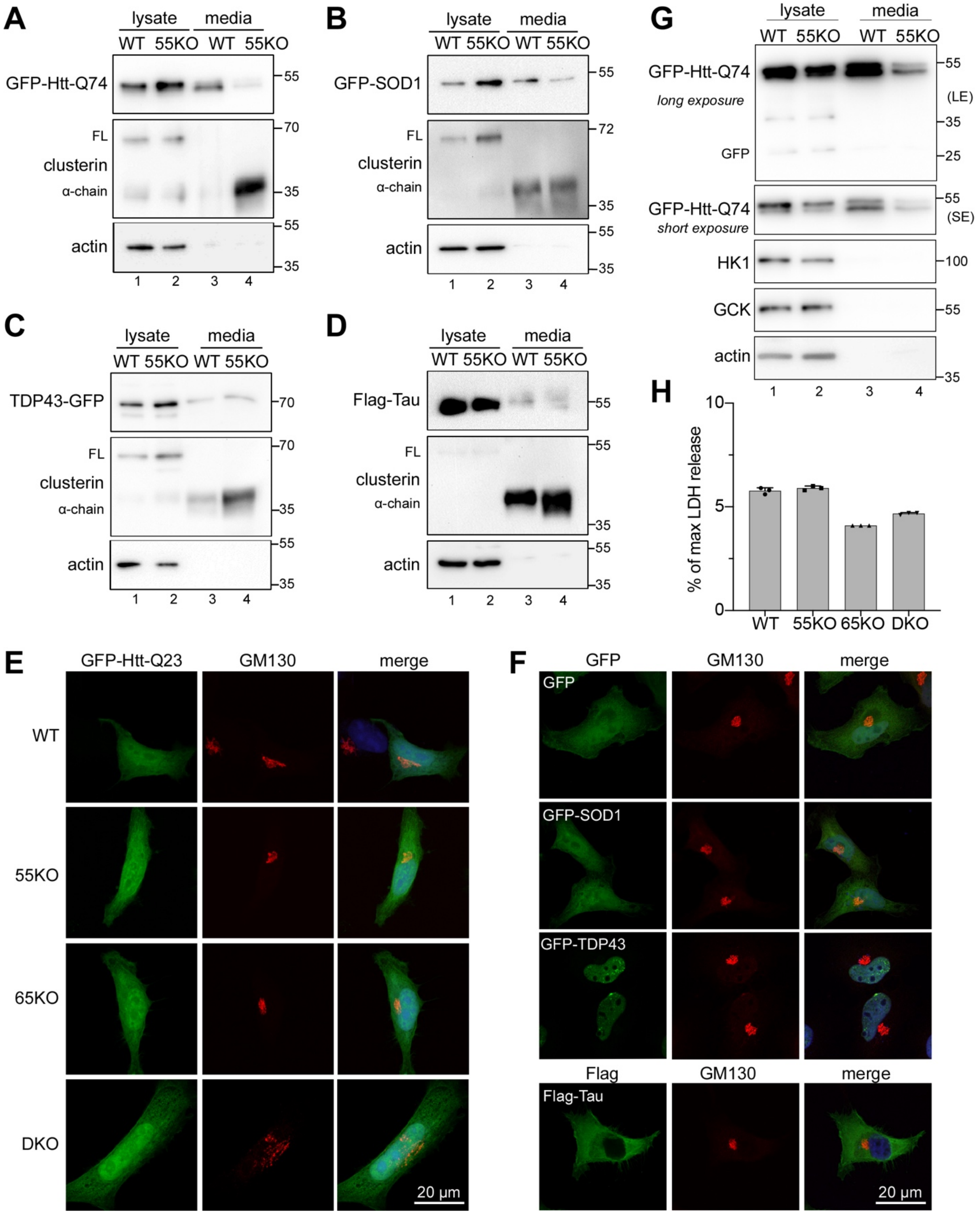

Figure S1. GRASP55 is required for the secretion of a subset of neurotoxic proteins. (A-B) Htt and SOD1 secretion is reduced by 55KO. WT and 55KO cells were transfected with GFP-Htt-Q74 (A) or GFP-SOD1 (B) and analyzed for secretion by western blot. Note that 55KO reduced the secretion of both Htt and SOD1. **(C-D)** TDP43 and Tau secretion is GRASP55 independent. WT and 55KO cells were transfected with TDP43-GFP (C) or Flag-Tau (D) followed by the analysis of TDP43 and Tau in the cell lysate and conditioned media. **(E)** GFP-Htt-Q23 expression does not disrupt the Golgi structure. GFP-Htt-Q23 was expressed in WT and GRASP KO cells and stained for GM130. The GM130 signal in DKO cells was increased to better visualize the Golgi morphology. **(F)** Expression of SOD1, TDP43 or Tau does not impact the Golgi structure. WT HeLa cells were transfected with indicated constructs and stained for GM130. **(G)** Htt is partially processed to produce free GFP. WT and 55KO cells were transfected with GFP-Htt-Q74 and analyzed for secretion by western blot. In addition to actin, two additional cytosolic proteins, hexokinase (HK1) and glucokinase (GCK), were blotted to exclude cell lysis. A long exposure of the GFP blot was shown to reveal an intermediate form of GFP-Htt-Q74 and free GFP, which indicates partial processing of GFP-Htt-Q74 in lysosomes prior to its secretion. **(H)** GRASP depletion does not cause cell death evaluated with a LDH cytotoxicity assay.

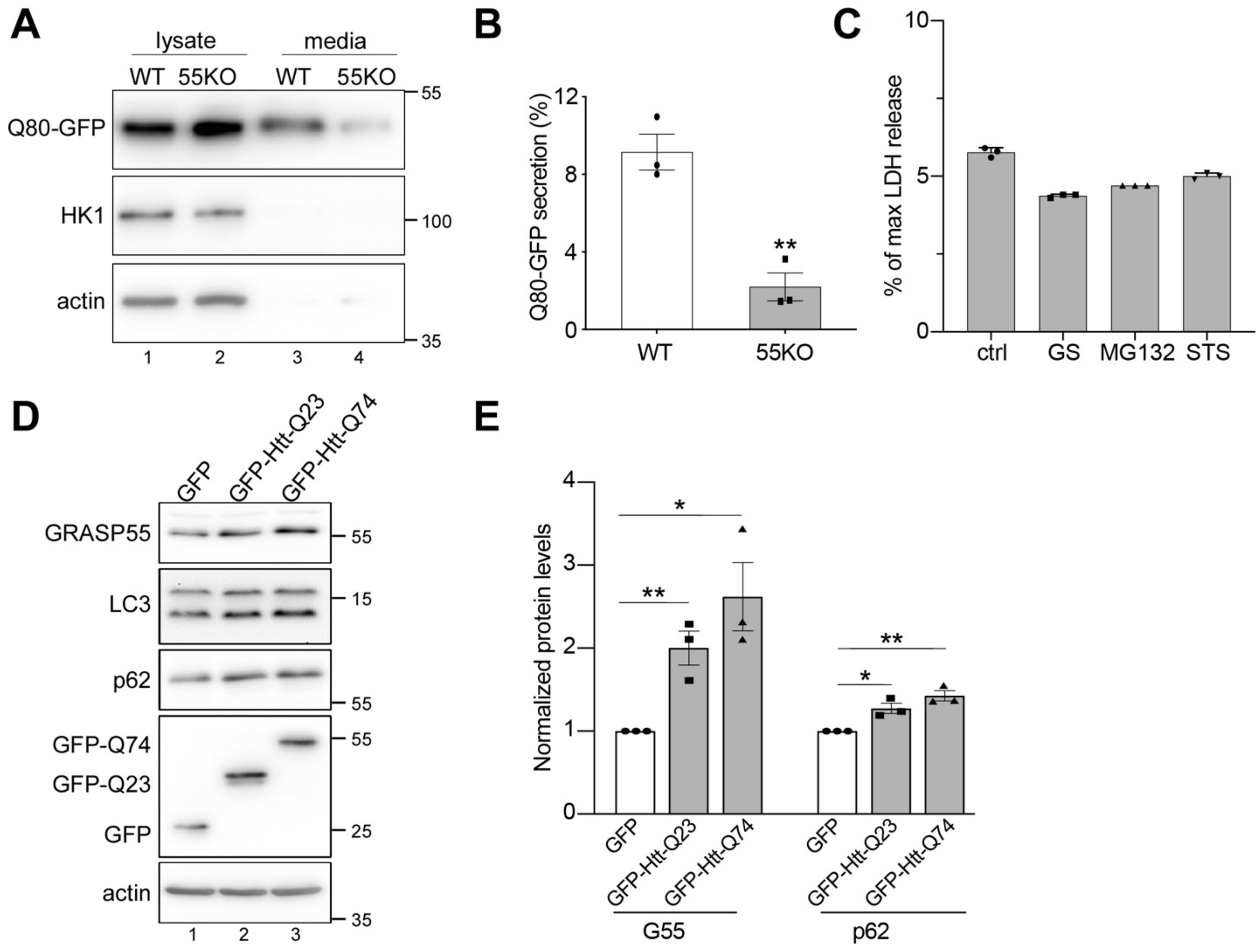

Figure S2. Q80-GFP is secreted via a GRASP55-dependent mechanism. (A) 55KO reduces Q80-GFP secretion. WT and 55KO cells were transfected with Q80-GFP and analyzed for secretion by western blot. Note that the percentage of secreted Q80 is similar to that of GFP-Htt-Q74, and that 55KO reduces the secretion of both polyQ and GFP-Htt-Q74 (compare the results with Figure 1C). **(B)** Quantification of Q80-GFP secretion in A. **(C)** Stress conditions used in this study do not cause cell death. WT HeLa cells were treated with glucose starvation (GS), 1 μM MG132, or 1 μM staurosporine (STS) for 4 h as indicated and analyzed by a LDH cytotoxicity assay. **(D)** GRAS55 level is upregulated by GFP-Htt-Q74 expression. WT cells expressing GFP, GFP-Htt-Q23 or GFP-Htt-Q74 were analyzed by western blot for indicated proteins. (**E**) Quantification of GRASP55 and p62 levels in D. Results are presented as mean ± SEM; statistical analysis was performed using Student's *t*-test. *, $p < 0.05$; **, $p < 0.01$.

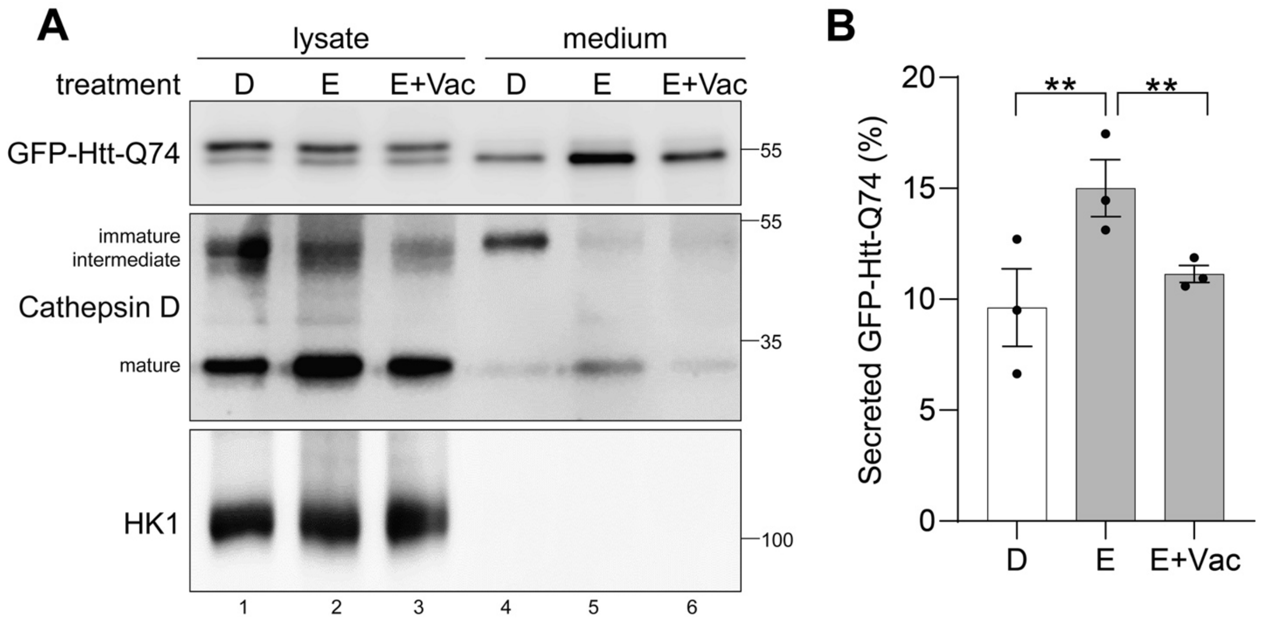

Figure S3. Inhibition of lysosome exocytosis by vacuolin-1 reduces Htt and mature cathepsin D secretion. A. HeLa cells transfected with GFP-Htt-Q74 were incubated in serum-free DMEM (D) or EBSS (E) with or without 5 μM vacuolin-1 for 4 h followed by western blot of Htt and cathepsin D in the cell lysates and conditioned media. Note that the secretion of both Htt and mature cathepsin D was enhanced by EBSS treatment, and this effect was abolished by vacuolin-1 treatment. **(B)** Quantification of Htt secretion in A.

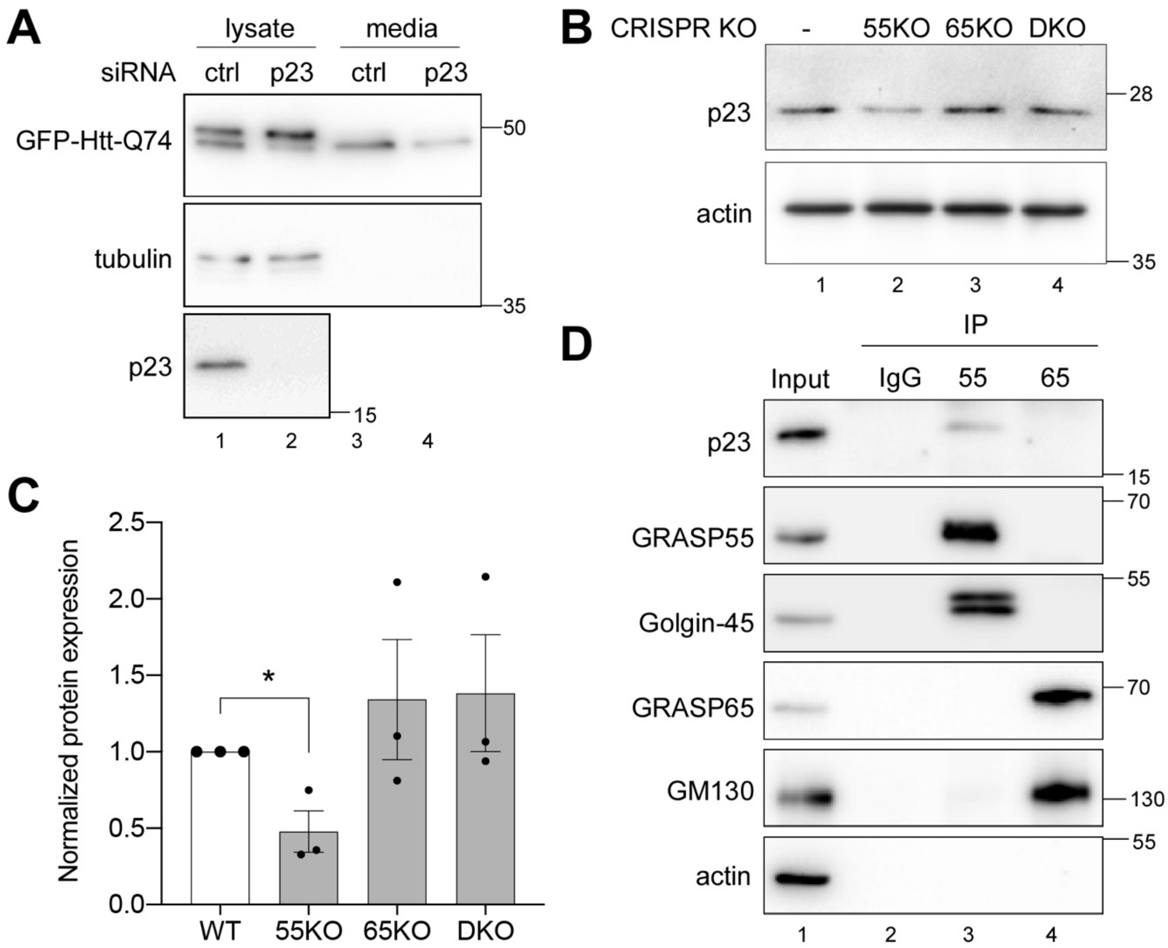

Figure S4. GRASP55 facilitates Htt secretion by stabilizing p23. (A) p23 KD reduces Htt secretion. WT HeLa cells were first transfected with control (ctrl) or p23 siRNA for 36 h and then transfected with GFP-Htt-Q74 for 24 h. Htt secretion was analyzed by western blot. **(B)** The p23 level is reduced in 55KO cells. Western blot analysis of p23 in WT and GRASP KO cells. **(C)** Quantitation of p23 in B. Results are presented as mean ± SEM; statistical analysis was performed using Student's *t*-test. *, $p<0.05$. **(D)** p23 interacts with GRASP55 but not GRASP65. Endogenous GRASP55 or GRASP65 was immunoprecipitated and their interaction with p23 was analyzed by western blot. Golgin-45 and GM130 were used as positive controls for GRASP55 and GRASP65, respectively.

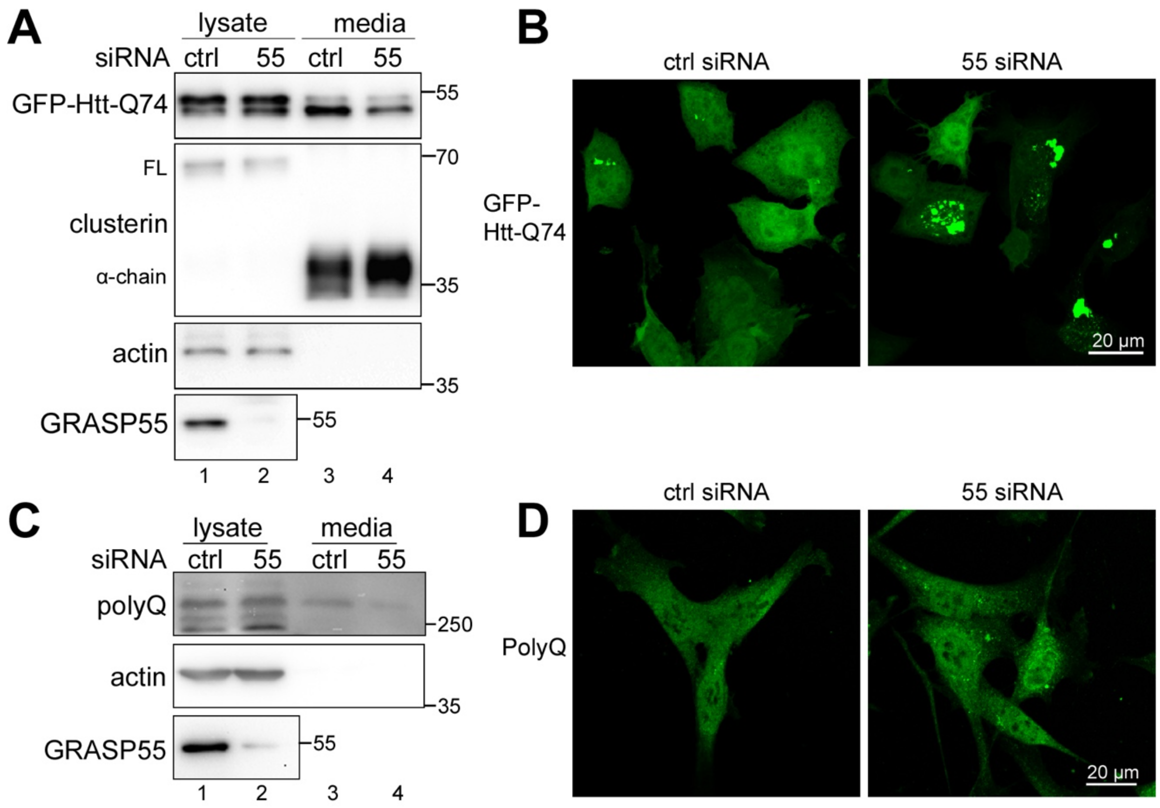

Figure S5. GRASP55 controls the secretion and aggregation of full length Htt-Q111 in striatal cells. (A-B) 55KD reduces Htt secretion but increases its aggregation in N2A cells. N2A cells were first transfected with control (ctrl) or GRASP55 siRNA for 36 h and then transfected with GFP-Htt-Q74 for 24 h. Htt secretion was analyzed by western blot (A) and its aggregation was analyzed by fluorescence microscopy (B). **(C-D)** 55KD reduces the secretion of full length Htt but increases its aggregation in ST*Hdh*Q111/Q111 cells. ST*Hdh*Q111/Q111 cells that express full length Htt-Q111 under the endogenous promotor were transfected with control (ctrl) or GRASP55 siRNA for 48 h and then incubated in Opti-MEM medium in the presence of a low dose (1 μM) of MG132 for 24 h. Cell lysates and conditioned media were analyzed by western blot for polyQ, actin and GRASP55 to show Htt secretion (C). Same cells incubated with full growth medium and 1 μM MG132 for 24 h were analyzed by fluorescence microscopy to show Htt aggregation (D).

Supplemental Tables

Table S1. List of differentially secreted proteins in 55KO vs. WT.

Gene names, fold changes [log2FC (55KO/WT media)], p value, and ER signal sequence are shown. Genes with significant change in 55KO compared to WT ($p < 0.05$) are manually selected and indicated whether they contain ER signal sequences. Data are sorted to rank the listed proteins based on the effect of 55KO on their secretion.

Table S2. List of selected GO term analysis of WT and 55KO secretome.

Secreted proteins with significant change in 55KO compared to WT ([|Log2FC (55KO/WT)|>0.5]; $p < 0.05$) are selected. Protein with or without ER signal sequence are separately processed in Metascape (https://metascape.org/gp/index.html#/main/step1) using the default setting. Top20 significant and relevant GO terms are selected out of Top100 GO terms.
